# Notes and News

**Published:** 1904-04-23

**Authors:** 


### NOTES AND NEWS.

LECTURES on tropical medicine are now given in the medical department of the University of Pennsylvania.

A BALL will be given at the Royal Albert Hall on June 9, under the presidency of Georgina, Lady Dudley, in aid of the fund for the removal of King's College Hospital to South London.

THE Duke of Marlborough will preside at a Festival Dinner in aid of the Alexandra Hospital for Children with Hip Disease, Queen Square, Bloomsbury, at Princes Restaurant, on Wednesday, April 27th.

THE result of the past year's work of the Hospital Saturday Fund may be regarded as very satisfactory. The collection amounted to £23,674, the largest yet received. The Lord Mayor will preside at the annual public meeting of the Fund on Saturday, April 23rd, at the Mansion House.

As the result of systematic inquiry, the executive committee appointed by the National Committee for the Establishment of a Sanatoria for Workers Suffering from Tuberculosis recommend the erection of a sanatorium for 200 patients. They estimate the cost at about £50,000 for building and site, and calculate that the cost of a patient would be about £65 per annum.

MESSRS. MARION, of Soho Square, not very long ago introduced a new process of reproducing facsimiles of oil-paintings in colours. They have gradually increased the collection of reproductions, the latest of which is that of the well-known picture by François Bruney entitled "À la Sauté du Chef." The picture is an example of rich and brilliant colouring, admirably adapted to demonstrate the excellence of this method of reproduction. The collection which Messrs. Marion are forming renders it possible to deck the walls of large rooms and institutions with first-class replicas of favourite pictures by the best artists, and this at the cost of an indifferent print.

THE apathy in Egypt in respect to the increasing amount of disease amongst cattle is very marked, and no effective means to stay the spread seem to be adopted.

THE Metropolitan Asylums Board will erect two additional infirmary blocks at the Tooting Bec Asylum, and a hall to accommodate about 300 patients. The cost is estimated at £26,250.

THE St. John's Ambulance Association holds an annual railway ambulance competition, and presents a challenge shield to the winners. The railways, on their part, hold a competition amongst their staff before this event to select its representatives.

MR. F. SWANZY, of the firm of Messrs. F. A. Swanzy, 147 Cannon Street, E.C., West African merchants, has been elected a member of the Committee of Management of the Seamen's Hospital Society. Mr. Swanzy's father was a prominent member of the Committee of the Seamen's Hospital Society in the days when the old *Dreadnought* floated in the river.

A NEWSPAPER war is being carried on at present over the question of the heating of railway carriages. It is quite surprising how many supporters the foot warmer finds. The foot warmer of uncertain temperature to begin with, a certain decreasing temperature when in use, and of nearly invariable difficulty of renewal. The foot warmer which imperils the shins of a passenger entering a full carriage creates envy and hatred amongst those who do not share its warmth, and is another means of encouraging the pernicious system of tipping.

THE Secretary of the League of Mercy informs us that T.R.H. the Prince and Princess of Wales, Grand President and Lady Grand President of the League of Mercy, have been pleased to appoint H.R.H. Princess Alexander of Teck Lady President of the Kingston-Surbiton District; the Duchess of Norfolk, Lady President of the Chichester District; the Viscount and Viscountess Gage, President and Lady President of the Lewes District; the Lord and Lady Boston, President and Lady President of the South Bucks District; the Lady Petre and Edward Murray Ind, Esq., Lady President and President of the Chelmsford District; Sir Harry Johnston, G.C.M.G., K.C.B., and the Hon. Lady Johnston, President and Lady President of the West St. Pancras District; the Hon. Mrs. Halford, Lady President of the Westminster District; Captain Sir Augustus F. Webster, Bart., and Lady Webster, President and Lady President of the Rye District; Mrs. Barrington Foote, Lady President of the Brentford District; Miss Cecilia E. Stone (Mayoress of Greenwich), Lady President of the Greenwich District; Dr. Whait, President of the Hampstead District; H. H. Riley-Smith, Esq., President of the West Newington District; Carl Hentschel, Esq., and Mrs. Carl Hentschel, President and Lady President of the Strand District; Mrs. Pim, Lady President of the Streatham District; and H. J. Strong, Esq., M.D., J.P., and Mrs. Philip Crick, President and Lady President of the Worthing District.

